# The mitochondrial fusion-associated protein MFN2 can be used as a novel prognostic molecule for clear cell renal cell carcinoma

**DOI:** 10.1186/s12885-023-11419-8

**Published:** 2023-10-16

**Authors:** Bin Zhang, Dali Han, LiMing Yang, Yang He, Shujun Yang, Hongbo Wang, Xingxing Zhang, Yuelin Du, Wei Xiong, Hualan Ha, Panfeng Shang

**Affiliations:** 1 Department of Urology, Institute of Urology, Key Laboratory of Urological Diseases in Gansu Province, Gansu Nephro-Urological Clinical Center, Lanzhou University Second Hospital, Lanzhou, 730030 Gansu China; 2 Department of Skin and Venereal Diseases, Jincheng People’s Hospital, Jincheng, 048000 Shanxi China

**Keywords:** MFN2, Clear cell renal cell carcinoma, Biomarker, Methylation, Immune infiltration, Bioinformatics

## Abstract

**Background:**

Mitofusin 2 (MFN2) plays an important role in many tumors, but how its role in renal clear cell carcinoma needs further research.

**Methods:**

In this study, we analyzed the expression of MFN2 in renal clear cell carcinoma tissues and normal kidney tissues through the Cancer Genome Atlas (TCGA) database and our clinical samples.Enrichment analysis was performed to determine MFN2-related pathways and biological functions. The correlation of MFN2 expression with immune cells was analyzed.The correlation of the expression of methylation and the methylation sites of MFN2 were analyzed by UALCAN and TCGA databases. Univariate / multivariate COX risk regression and Kaplan-Meier methods were used to determine the prognostic value of MFN2.Nomograms were drawn to predict overall survival (OS) at 1,3, and 5 years. We investigated the role of MFN2 in renal cancer cells using CCK 8, clone formation, wound healing assay, and methylase qPCR experiments.

**Results:**

MFN2 is poorly expressed in renal clear cell carcinoma compared to normal kidney tissue,and is significantly negatively associated with TNM stage, histological grade and pathological stage.MFN2 was directly associated with OS after multivariate Cox regression analysis.MFN2 shows a hypomethylation state and shows a positive correlation with multiple methylation sites.Signaling pathways through functional enrichment to B-cell receptors and oxidative stress-induced senescence.Moreover, the low expression of MFN2 was positively correlated with the degree of immune cell infiltration in a variety of immune cells.In vitro experiments showed that overexpression of MFN2 significantly inhibited the proliferation and migration of renal clear cells and promoted methylation.

**Conclusions:**

In conclusion, MFN2 can be used as a novel prognostic marker for renal clear cell carcinoma and requires further investigation of its role in tumor development.

**Supplementary Information:**

The online version contains supplementary material available at 10.1186/s12885-023-11419-8.

## Background

Renal cell carcinoma (RCC) is considered to be one of the most frequent urological malignancies threatening human health worldwide, with new data showing 76,080 new cases in 2021, including 13,780 cases of death [1].The incidence of renal cell carcinoma (RCC) is 3.8% of all new tumors throughout the year [2], with clear cell renal cell carcinoma being up to 70% [3–5].The preferred treatment for RCC is surgical treatment, although, distant metastasis occurs in 20–30% of patients, and the 5-year mortality of postoperative RCC is up to 45% [6, 7].Due to the different activities of the tumor cells, a completely different prognosis ofresults in it [8].In order to more accurately understand the cellular activity of RCC, it is urgent to find out the molecular markers leading to the clinical prognosis of patients, and to provide more precise treatment for patients.

Mitochondria play an important role in cellular metabolism, mainly supplying cell energy through oxidative phosphorylation, and they are in the dynamic process of mitochondrial division and fusion [9].Such a dynamic process is essential in maintaining mitochondrial morphology and function [10].Mitofusin 2 is mainly found on the outside mitochondrial membrane and its main function is to participate in mitochondrial fusion and maintain mitochondrial homeostasis and activity [11].Multiple studies have shown that MFN2 expression is associated in the development of multiple cancers.For example, MFN2 expression in lung cancer [12], hepatocellular carcinoma [13],gastric cancer [14],and colorectal cancer [15] are lower than normal tissues, and it plays a role in inhibiting tumor growth.It has been shown that knockdown of MFN2 in MCF7 and A549 cells promotes tumor invasion and migration and promoted proliferative [16].In bladder cancer, cell cycle arrest and promoted apoptosis occurred after MFN2 overexpression [17].It can be suggested that MFN2 can act as a oncogenic inhibitory factor and act as a new molecular marker in tumors.But currently the current role of MFN2 in ccRCC remains unknown. Therefore, we explored for the first time how MFN2 functions in ccRCC through the TCGA database.

Thanks to the rapid development of high-throughput sequencing technologies, we can currently investigate those molecular markers of clinical and prognostic significance through bioinformatics studies [18].In this study, the analysis of the TCGA database has obtained the analysis of MFN2 expression in ccRCC and its prognostic role in OS, and further analyzed the correlation of MFN2 expression with methylation and immunity.This study helps to provide new therapeutic ideas and methods for the therapy of clear cell renal cell carcinoma.

## Methods

### Data acquire and analyse

We analysed the TCGA database^1^ (including 72 adjacent tissue data 531 cancer tissue data) and the GTEx database^2^ (including 28 normal tissue data) for clinical data information and mRNA expression matrices from ccRCC patients.The other two GEO datasets are GSE14762 (including 12 normal tissues and 10 tumor tissues) and GSE53757 (including 72 normal tissues and 72 tumor tissues).The obtained FPKM data were normalized to the TPM data for the pan-cancer analysis and the mRNA expression analysis of MFN2, respectively.We again overlapped MFN2 mRNA to obtain genetic and clinical information on MFN2.We used OS as our primary outcome and calculated differentially expressed genes (DEGs) with the ‘limma’ package at adjusted P < 0.05 & | log2 fold change|≥1.We used the ‘survminer’ package for visualisation and the ‘surviva’ package for statistical analysis of survival data [19].

(1 https://portal.gdc.cancer.gov/,2https://commonfund.nih.gov/gtex)

### Analysis of the differentially expressed genes

By the expression of MFN2 in the TCGA database, it was separated into high expression and low expression groups by median expression value [20].Two sets of data were used for differentially expressed gene (DEG) analysis with the DESeq2 of the R package, and the DEG threshold was set to adjusted p value < 0.05, |log2-fold-change (FC)|>1.Heatmaps of the correlation between the top 10 DEG expression and MFN2 were made by the Spearman correlation analysis.

### Functional enrichment analysis

We performed a GO and KEGG analysis of the DEGs data [21], using the toolkit for the R package GOplot [22].We used R package cluster analysis for GSEA analysis [23, 24], with the conditions for the functional enrichment and pathway enrichment considered significant in the analysis results were P. adjust < 0.05 and FDR < 0.25.

### DNA methylation analysis

To investigate the possible mechanisms of MFN2 in renal clear cell carcinoma, we assessed MFN2 methylation levels and its prognostic value using the MethSurv database^3^ [25]. In addition we analysed the MFN2 promoter methylation status by using the UALCAN database^4^ [26].

(3 https://biit.cs.ut.ee/methsurv/, 4 http://ualcan.path.uab.edu)

### Immune infiltration analysis

We used 17 immune cells for immune level calculation and a single sample GSEA to clarify the enrichment score of such immune cells among renal clear cell carcinomas, completed by R package GSVA [27].The expression of MFN2 was analysed by Spearman’s correlation analysis with immune cells and the Wilcoxon ranksum test was applied to analyse the difference in the level of immune infiltration among the high and low MFN2 expression groups.

### Baseline characteristics and survival prognosis analysis

Download the RNAseq data in the level 3 HTSeq-FPKM format from the TCGA (https://portal.gdc.cancer.gov/) KIRC (Kidney Clear Cell Cancer) project.The RNAseq data in the FPKM (Fregments Per Kilobase per Million) format was converted into the TPM (transcripts per million reads) format and was log2 transformed.The median expression value of MFN2 was taken as the cut-off value and Splited into MFN2 high expression group and MFN2 low expression groups to further analyze the relationship between the clinicopathological characteristics of MFN2 high expression group and low expression group. Survival analysis was performed using the Kaplan – Meier method and the lon-rank test.

### Univariate/multivariate Cox risk regression analysis and nomogram construction

To obtain independent prognostic factors associated with ccRCC and OS, we performed univariate COX regression with MFN2 and five clinical features, and parameters with P-values<0.001 for multivariate COX risk regression, using the R package survival package(version 3.2–10)for statistical analysis of survival data.In addition, we used the R” RMS “and” sesivalROC " packages to establish the nomograms, and used the calibration curves to evaluate the performance of the nomogram, and used the C-index to quantify the identification of the nomograms.

### Analysis of the interaction network between protein and protein

The STRING database^5^ was used to query genes interacting with the MFN2 gene and for PPI network building, with confidence values > 0.7 and default for other parameters, and Cytoscape software^6^ was used to visualise the PPI network [28].To recognize the first 10 reciprocal genes of the DEG, we used the CytoHubba plugin in Cytoscape. A heat map of gene co-expression was further made. Scatterplot of genes positively associated between MFN2 and the top 10 hub genes based on gene expression, with a P-value < 0.05.

(5 http://string-db.org/ 6 https://cytoscape.org/)

### Human tissue and cell culture

This study collected carcinoma and paired adjacent tissues from six patients undergoing renal clear cell carcinoma surgery at Lanzhou University Second Hospital from January 2020 to June 2020.The ethics of tissue specimens was reviewed and endorsed by the Ethics Committee of the Lanzhou University Second Hospital, and each patient enrolled in the group was given information and signed a consent form(Approval Number: 2023 A-268).The cell lines (HK2,7860, CAKI-1, and A498 cells) used in this study were all offered by the Gansu Provincial Key Laboratory of Urological Diseases.The HK2 cells were cultured using DMEM / F12 medium (Gibco) supplemented with 10% fetal bovine serum (PAN-Biotech GmbH),7860, CAKI cells were cultured using 1640 medium (basalmedia) supplemented with 10% fetal bovine serum (PAN-Biotech GmbH),the A498 cells were cultured using MEM medium (Gibco) supplemented with 10% fetal bovine serum (PAN-Biotech GmbH),all cells were cultured in 5%CO2 at 37℃.

### Pathological sample obtain

Samples of 20 pairs of renal clear cell carcinoma and adjacent tissues were collected and embedded in the Department of Pathology of Lanzhou University Second Hospital from January 2018 to December 2019. This study was approved by the Ethics Committee of the Lanzhou University Second Hospital(Approval Number:2023 A-268).

### Immunohistochemistry

The obtained tumor carcinoma and adjacent tissues were first fixed in 10% formalin, paraffin-embedded and then sectioned, and placed on glass slides.Then, after dewaxing, hydration, and sealing, the slides were incubated with a 1:200 dilution of MFN2 antibody (proteintech, Cat No.12186-1-AP) for 4℃ overnight.The next day, secondary antibodies were incubated at room temperature for 30 min, colored by DAB for 15 min, counterstained with hematoxylin, dehydrated, and sealed.

### Quantitative real-time PCR analysis

We selected TRIzol reagent to extract total RNA from each cell line, normalized RNA concentration by spectrophotometry before reverse transcription, 1ug of total RNA was reverse transcribed as cDNA according to the AJ reverse transcription kit instructions, and qRT-PCR was performed with BIO-RAD CFX-96 and calculated by 2^−ΔΔCt^method.Statistical analysis and mapping were performed with Prism8.0.The primers used in this study include:MFN2,F:5’-GCAGAAGGCTTTCAAGTGAGGAT-3’;R:5’-GGTCTTGCCGCTCTTCACG-3’;FTO,F:5’-GCCCGAACATTACCTGCTG-3’;R:5’-TGCTCCTTCTAGGGTTTTGCT-3’;ALKBH5,F:5’-CGGCGAAGGCTACACTTACG-3’;R:5’-CCACCAGCTTTTGGATCACCA-3’;METTL3,F:5’-CAAGCTGCACTTCAGACGAA-3’;R:5’-GCTTGGCGTGGTCTTT-3’;METTL14,F:5’-TGGTTCAAGTGACACTACCA-3’;R:5’-TTGGTTGGACTACTTTCTGCTA-3’;Actin,F:5’-CAGTCGGTTGGAGCGAGCAT-3’;R:5’-TGGCTTTTAGGATGGCAAGGGAC-3’.

### Western blot assay

Total proteins from the surgically removed carcinoma tissues and the corresponding adjacent tissues from 6 pairs of ccRCC patients and those from each cell line were extracted, respectively.The protein concentration was determined using the BCA Protein Concentration Assay Kit (Solarbio).According to the protein concentration, 20ug of total protein was electrophoresis on a 10% SDS-PAGE gel, and after electrophoresis the corresponding protein was electrotransferred onto a 0.45 m PVDF membrane (Biosharp).After shaking the membrane in 5% skim milk powder at room temperature for 1 h, the membrane was washed with TBST three times for 5 min each.Incubate the membrane overnight at 4 °C in primary antibody MFN2 (rabbit 1:1000, Proteintech, 12186-1-AP), β-actin (rabbit 1:2000, Proteintech, 20536-1-AP).The next day, secondary antibodies (HRP Goat Anti-Rabbit 1:10000, ABclonal, AS014) were incubated at room temperature, washed three times with TBST for 10 min each, and exposed with an ECL chemiluminescence kit (Beyotime).

### Transfection of cells

We selected renal clear cell carcinoma cells 786-O and Caki-1 for cell transfection.The MFN2 lentiviral overexpression vector was designed and synthesized by Hanheng Biology (Shanghai, China).We first used 96-well plates to explore the optimal MOI values for both the 786-O and Caki-1 cells.We separately spread 1*10^4^ cells into the six-well plates and incubated them in 37℃ and 5% CO_2_ overnight.For transfection the next day, the added viral amounts of 786-O cells were calculated at MOI = 20 and Caki-1 at MOI = 30 for transfection at a polybrene concentration of 4 u g/ml.After 24 h of transfection, the medium containing the virus was aspirated and replaced with fresh complete medium.The transfection efficiency was observed by inverted fluorescence microscopy after 48 h of transfection. After 72 h of transfection 2ug/ml of puromycin was added to 786-O cells and 1ug/ml of puromycin was added to Caki-1 cells for screening of stable transfectants.After successful screening of the stable transgenic cells, we verified the overexpression efficiency by PCR and WB experiments and performed the next functional experiments.

### Proliferation experiments

Successfully transfected 786-O and Caki-1 cells were added 3*10^3^ cells to each well of a 96-well plate divided into 0 h, 24 h, 48 h, 72 and 96 h groups according to the grouping of LV-MOCK and LV-OE-MFN2, respectively. After the cells were plastered, 90 ul of complete medium + 10ul of CCK 8 solution (biosharp, Anhui, China) was added per well.After being placed in a cell incubator at 37 °C for 30 min, the absorbance values were monitored separately at 450 nm. Three replicates of each experiment were performed and data were analysed and plotted using Prism8.

### Clone formation

Successfully transfected 786-O and Caki-1 cells were added 1*10^3^ cells each to 6-well plates grouped according to LV-MOCK and LV-OE-MFN2, respectively. The 6-well plates were incubated in a cell incubator at 37 °C with 5% CO_2_ and the medium was changed regularly. After 10–15 days of incubation, the culture was terminated when more than 50 cells per well were observed to be monoclonal, the medium was aspirated, PBS was added and washed gently three times, 1ml of paraformaldehyde was added to each well and fixed for 15–30 min, the fixative was aspirated and then 0.1% crystalline violet was added for 20 min, then repeatedly with PBS for three times.After the 6-well plates had dried, photographic counts were taken and the data analysed and plotted using Prism8.

### Wound-healing assay

Successfully transfected 786-O and Caki-1 cells were added 5*10^5^ cells to 6-well plates according to the grouping of LV-MOCK and LV-OE-MFN2, respectively, and incubated overnight in a 37 °C, 5% CO_2_ cell culture incubator.A 200 ul sterile pipette tip was used for the scratching experiments. After scratching, serum-free medium was added after three gentle washes with PBS and photographed.Place in a 37 °C, 5% CO2 cell incubator for 24 h and then photograph. Analysis was performed using ImageJ.

## Results

### Clinical features of the patients

Our cohort contains clinical information and RNA sequencing data from the TCGA database of 603 patients with renal clear cell carcinoma, including 72 patients with renal clear cell carcinoma with their paired paracancerous tissue samples. In supplement to this, we also downloaded sequencing data from the GTEx database for 28 normal kidney tissues to expand the sample size of normal kidney tissues. The clinicopathological features are shown in Table 1.

**Table 1:** Clinicopathological characteristics of high- and low-MFN2 expression groups

Characteristic	Low expression of MFN2	High expression of MFN2	p
n	269	270	
Age, n (%)			0.413
<=60	129 (23.9%)	140 (26%)	
> 60	140 (26%)	130 (24.1%)	
Gender, n (%)			**< 0.001**
Female	71 (13.2%)	115 (21.3%)	
Male	198 (36.7%)	155 (28.8%)	
T stage, n (%)			**< 0.001**
T1	114 (21.2%)	164 (30.4%)	
T2	40 (7.4%)	31 (5.8%)	
T3	110 (20.4%)	69 (12.8%)	
T4	5 (0.9%)	6 (1.1%)	
N stage, n (%)			0.859
N0	122 (47.5%)	119 (46.3%)	
N1	9 (3.5%)	7 (2.7%)	
M stage, n (%)			**0.008**
M0	207 (40.9%)	221 (43.7%)	
M1	51 (10.1%)	27 (5.3%)	
Pathologic stage, n (%)			< 0.001
Stage I	110 (20.5%)	162 (30.2%)	
Stage II	30 (5.6%)	29 (5.4%)	
Stage III	74 (13.8%)	49 (9.1%)	
Stage IV	53 (9.9%)	29 (5.4%)	
Histologic grade, n (%)			**0.001**
G1	3 (0.6%)	11 (2.1%)	
G2	105 (19.8%)	130 (24.5%)	
G3	108 (20.3%)	99 (18.6%)	
G4	50 (9.4%)	25 (4.7%)	
OS event, n (%)			**< 0.001**
Alive	154 (28.6%)	212 (39.3%)	
Dead	115 (21.3%)	58 (10.8%)	
DSS event, n (%)			**< 0.001**
Alive	183 (34.7%)	237 (44.9%)	
Dead	82 (15.5%)	26 (4.9%)	
PFI event, n (%)			**< 0.001**
Alive	158 (29.3%)	220 (40.8%)	
Dead	111 (20.6%)	50 (9.3%)	

### Reduced expression of MFN2 in renal clear cell carcinoma

**Fig. 1:**
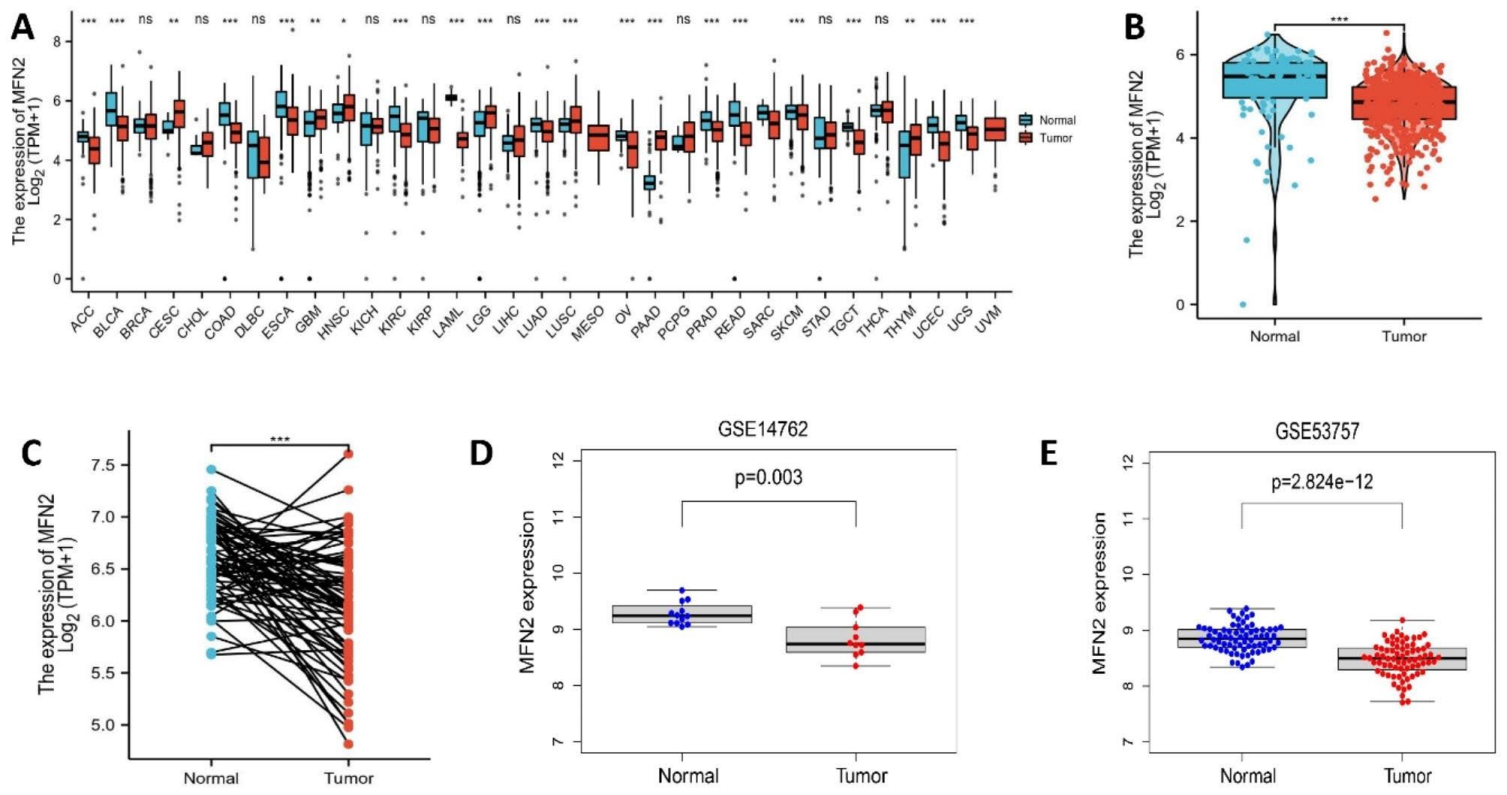
Reduced expression of MFN2 in renal clear cell carcinoma. (**A**) Pan-cancer analysis showed low MFN2 expression in most tumors. (**B**) MFN2 expression was significantly lower in clear cell renal cell carcinoma tissue relative to normal kidney tissue (p < 0.001). (**C**) MFN2 showed low expression in 72 matched renal clear cell carcinoma samples (P < 0.001). (**D**) MFN2 was lowly expression in renal clear cell carcinoma tissues in dataset GSE14762 (P = 0.003). (**E**) MFN2 was lowly expression in renal clear cell carcinoma tissues in dataset GSE53757 (P = 2.824e-12)

MFN2 expression showed low expression in most tumours, such as: bladder cancer, renal clear cell carcinoma, colon cancer, prostate cancer and rectal adenocarcinoma (Fig. 1A).MFN2 expression was significantly lower in clear cell renal cell carcinoma tissue relative to normal kidney tissue (p < 0.001) (Fig. 1B).MFN2 showed low expression in 72 matched renal clear cell carcinoma samples (P < 0.001) (Fig. 1C).We also did validation in two GEO datasets and revealed that MFN2 was lowly expression in renal clear cell carcinoma tissues in dataset GSE14762 (P = 0.003) (Fig. 1D) and in dataset GSE53757 (P = 2.824e-12) (Fig. 1E).

**Fig. 2:**
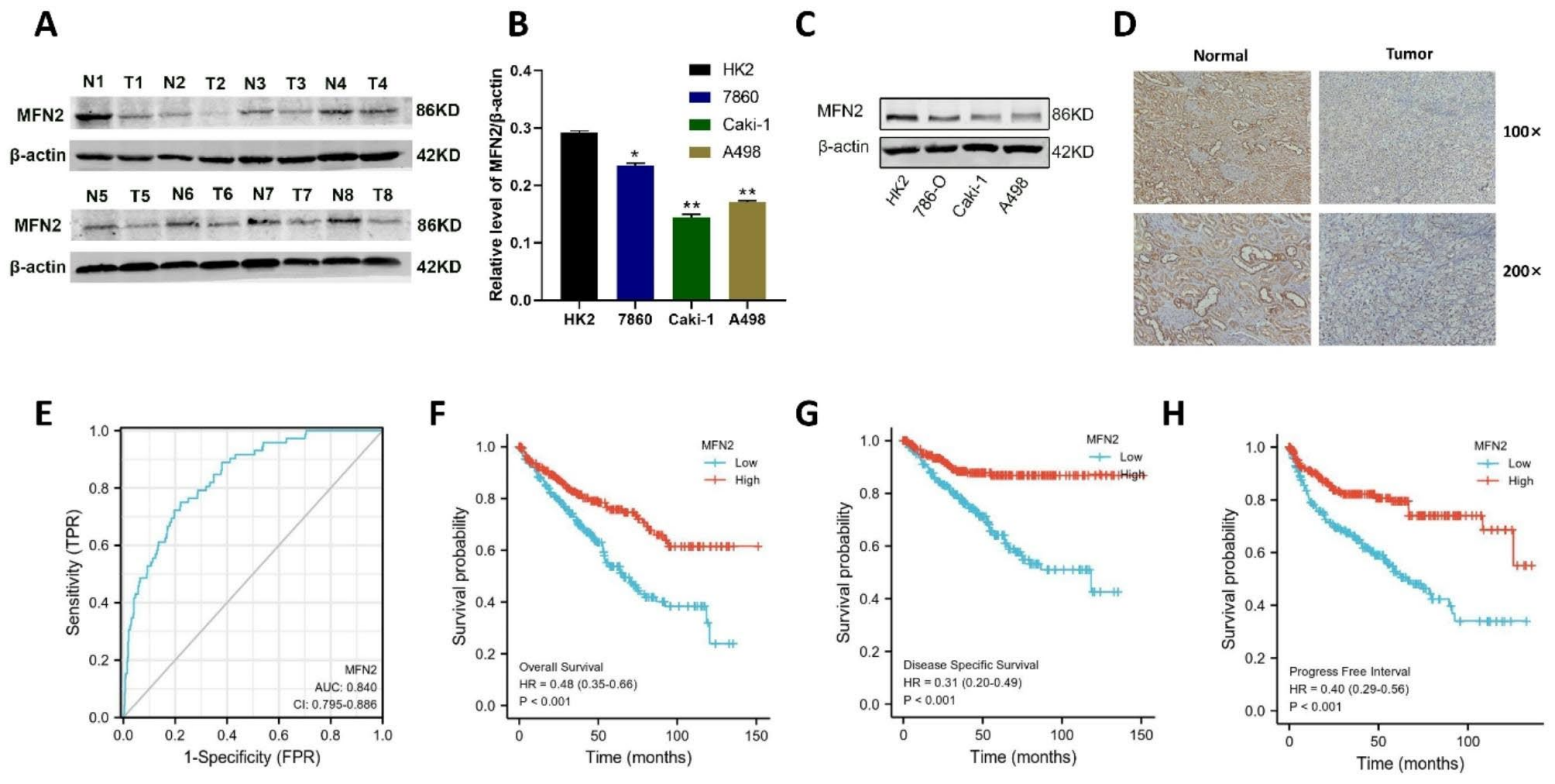
The MFN2 low expression was observed in renal clear cell carcinoma tissues and cells, and the low expression of MFN2 was associated with a worse prognosis. (**A**) MFN2 low expression in eight pairs of primary renal clear cell carcinoma tissues. (**B–C**) MFN 2 showed low expression at both PCR and WB levels in renal clear-cell carcinoma cells 786O, Caki-1, and A498 relative to normal renal tubular epithelial cells HK2. (**D**) Immunohistochemistry results showed that MFN2 expression in renal cancer tissues was significantly lower than that in adjacent tissues. (**E**) The ROC curve verified that MFN2 expression had good predictive power for renal clear cell carcinoma. (AUC = 0.840, CI = 0.795−0.886). (**F–H**) KM survival analysis showed that patients with renal clear cell carcinoma in the MFN2 low expression group had poorer OS, DSS and PFI than the high expression group (P < 0.001)

To further clarify the expression of MFN2, we extracted total protein from eight pairs of primary renal clear cell carcinoma and paraneoplastic tissues in our hospital, and the results of westernblot showed low expression in all renal clear cell carcinomas (Fig. 2A).In addition we verified that MFN2 expression in renal clear cell carcinoma cells 786O, Caki-1, and A498 was suggestive of low expression at both PCR and WB levels relative to MFN2 expression in normal renal tubular epithelial cells (Fig. 2B-C).Immunohistochemistry results showed that MFN2 expression in renal cancer tissues was significantly lower than that in adjacent tissues (Fig. 2D).The ROC curve verified that MFN2 expression had good predictive power for renal clear cell carcinoma and the area below the curve (AUC) was 0.840 (95% confidence interval [CI] = 0.795–0.886) (Fig. 2E).Using the median expression of MFN2 as the cut-off value, KM survival analysis showed that patients with renal clear cell carcinoma in the MFN2 low expression group had poorer overall survival than the high expression group (P < 0.001) (Fig. 2F), patients with renal clear cell carcinoma in the MFN2 lower expressing group showed poorer disease specific survival than in the higher expressing group (P < 0.001) (Fig. 2G), and patients with renal clear cell carcinoma patients had poorer tumor progress free interval than the high expression group (P < 0.001) (Fig. 2H).

### The relationship between MFN2 expression and clinicopathological factors

**Fig. 3:**
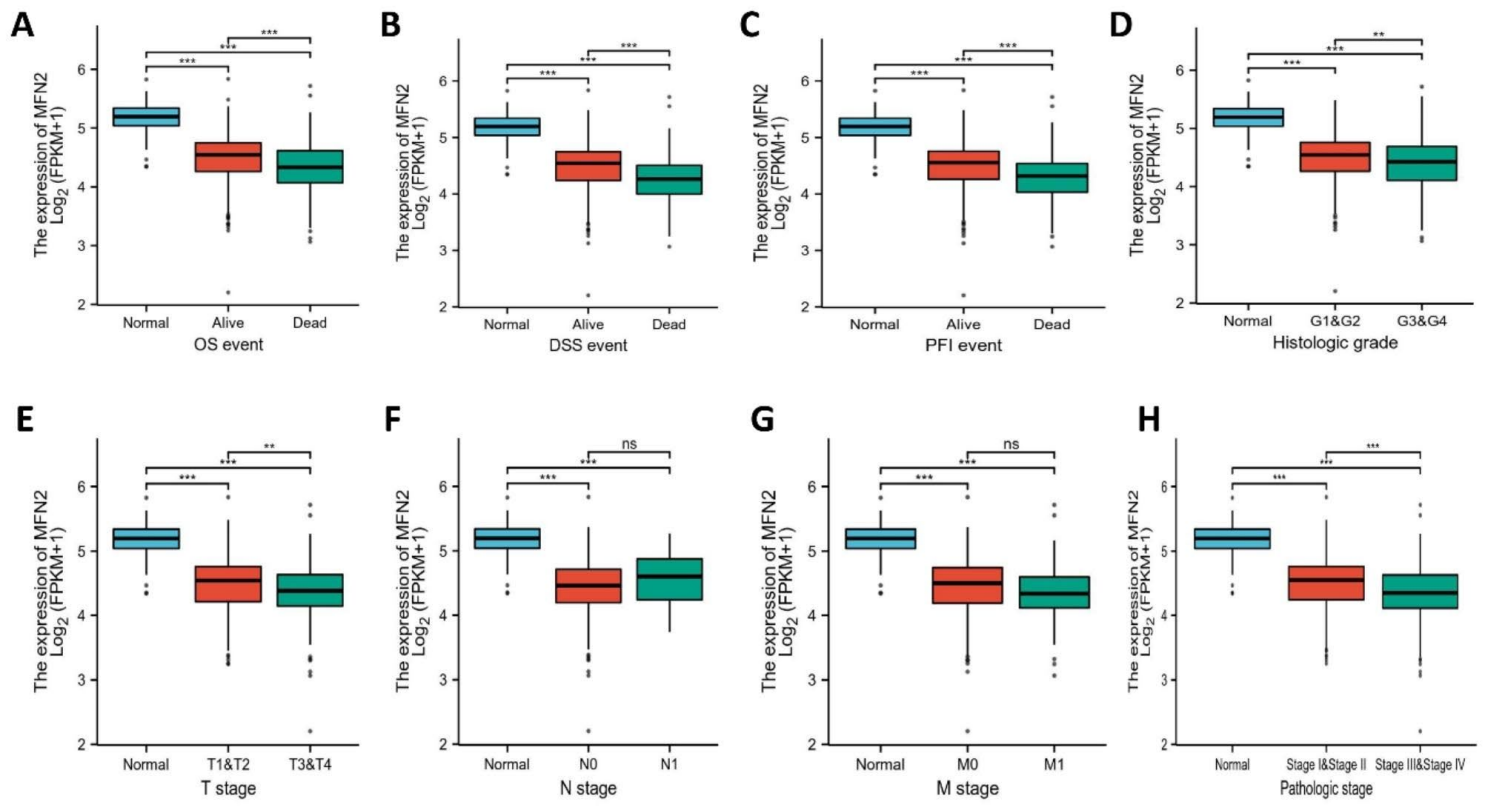
The expression of MFN2 was significantly associated with clinicopathological factors. Data are shown for (**A**) Overall survival (OS) event (P < 0.001). (**B**) Disease-specific survival (DSS) event (P < 0.001). (**C**) Progress free interval (PFI) event (P < 0.001). (**D**) Histologic grading (P < 0.01). (**E**) T stage (P < 0.01). (**F**) N stage (P = 0.859). (**G**) M stage. (**H**) pathologic stage (P < 0.001)

In Fig. 3, low expression of MFN2 was significantly associated with histologic grading (P < 0.01), pathologic stage (P < 0.001), T stage (P < 0.01), overall survival (OS) event(P < 0.001), disease-specific survival (DSS) event (P < 0.001) and disease-free progression interval of survival (PFI) event (P < 0.001).In Table 2, univariate logistic regression analysis indicated some differences among groups with high and low MFN2 expression and clinicopathology, including gender (OR = 0.483, 95% CI = 0.335–0.693, P < 0.01), T stage (OR = 0.515, 95% CI = 0.359–0.736, P < 0.01), M stage (OR = 0.496, 95% CI = 0.296–0.814, P = 0.006), pathological stage (OR = 0.450, 95% CI = 0.314–0.642, P < 0.001) and histologic grade (OR = 0.601, 95% CI = 0.426–0.847, P = 0.004).

**Table 2:** Associations of MFN2 expression with clinicopathological characteristics of patients (n = 539)

Characteristics	Total(N)	Odds Ratio(OR)	P value
T stage (T3&T4 vs. T1&T2)	539	0.515 (0.359–0.736)	**< 0.001**
N stage (N1 vs. N0)	257	0.797 (0.277–2.208)	0.663
M stage (M1 vs. M0)	506	0.496 (0.296–0.814)	**0.006**
Gender (Male vs. Female)	539	0.483 (0.335–0.693)	**< 0.001**
Age (> 60 vs. <=60)	539	0.856 (0.610–1.199)	0.366
Pathologic stage (Stage III&Stage IV vs. Stage I&Stage II)	536	0.450 (0.314–0.642)	**< 0.001**
Histologic grade (G3&G4 vs. G1&G2)	531	0.601 (0.426–0.847)	**0.004**

### Identification of DEGs in clear cell renal cell carcinoma

**Fig. 4:**
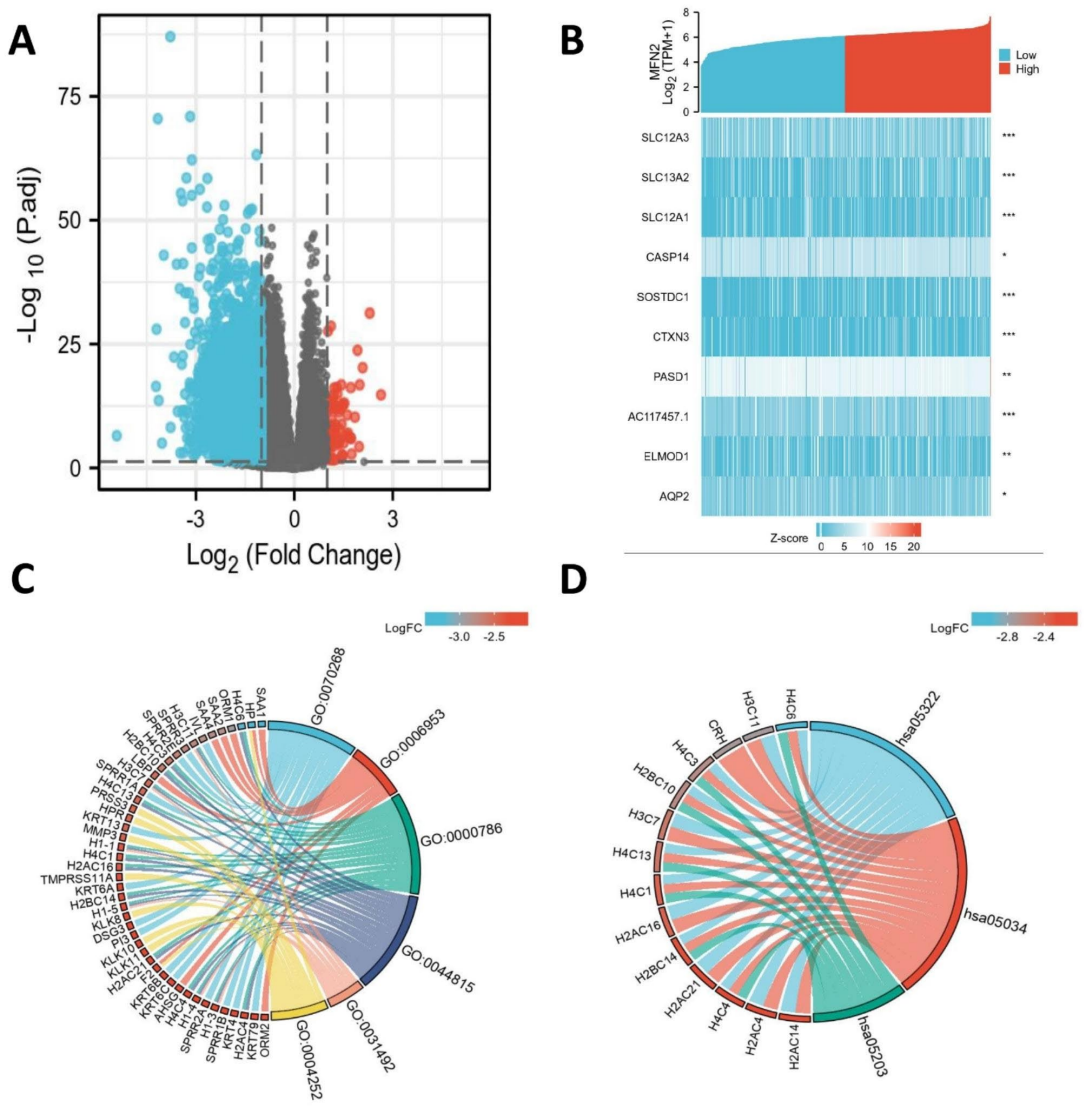
MFN2-related differentially expressed genes (DEGs) and GO, KEGG functional enrichment analysis in clear cell renal cell carcinoma. (**A**) Volcano plot of DEGs. Blue and red dots indicate the significantly down-regulated and up-regulated DEGs, respectively. (**B**) Heatmap of correlation between MFN2 expression and the top 10 DEGs. (**C**) GO analysis of DEGs. (**D**) KEGG analysis of DEGs. GO, Gene Ontology; KEGG, Kyoto Encyclopedia of Genes and Genomes; DEGs,differentially expressed genes. *p < 0.05, **p < 0.01, and ***p < 0.001

A total of 9075 differential genes were screened in the MFN2 high and low expression groups, including 81 up-regulated DEGs and 8994 down-regulated DEGs (P.adjust < 0.05, |Log2-FC|>1) (Fig. 4A ).We use a correlation heat map to represent the relationship of MFN2 with the first 10 DEGs(including SLC12A3, SLC13A2, SLC12A1, CASP14, SOSTDC1, CTXN3, PASD1, AC117457.1, ELMOD1, AQP2)(Fig. 4B and Supplementary Table S 1).

### GO, KEGG and GSEA functional enrichment analysis

**Fig. 5:**
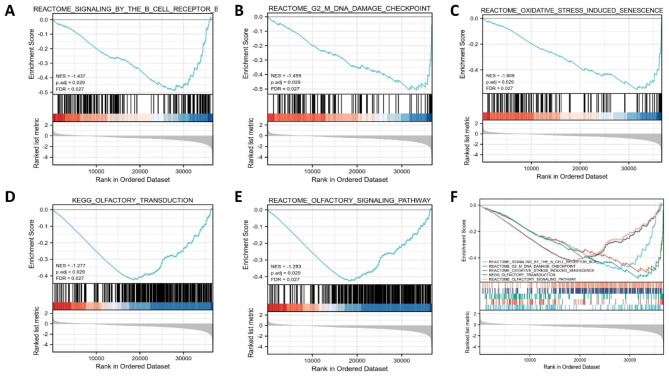
Gene set enrichment analysis (GSEA) of DEGs. (**A**) B-cell receptors pathway. (**B**) G2-M DNA damage checkpoints pathway. (**C**) Oxidative stress-induced senescence pathway. (**D**) Olfactory transduction pathway. (**E**) Olfactory signaling pathway. (**F**) All of these five eligible signaling pathways significantly enriched in the low MFN2 expression phenotype

GO functional enrichment analysis encompassed three aspects of biological processes, cellular composition and molecular function, and GO analysis of DEGs revealed e.g. cornification,acute-phase response,nucleosome, DNA packaging complex, nucleosomal DNA binding and serine-type endopeptidase activity (Fig. 4C and Supplementary Table S 2).Analysis of the KEGG pathway in DEGs indicated enrichment to systemic lupus erythematosus, alcoholism, and viral carcinogenesis (Fig. 4D and Supplementary Table 3). We then performed GSEA analysis of the MFN2 high and low expressing groups and found that the MFN2 low expressing group was notably enriched in multiple signalling pathways such as G2-M DNA damage checkpoints, B-cell receptors, olfactory transduction, and oxidative stress-induced senescence (Fig. 5A-F).

### Correlation between MFN2 expression and methylation

**Fig. 6:**
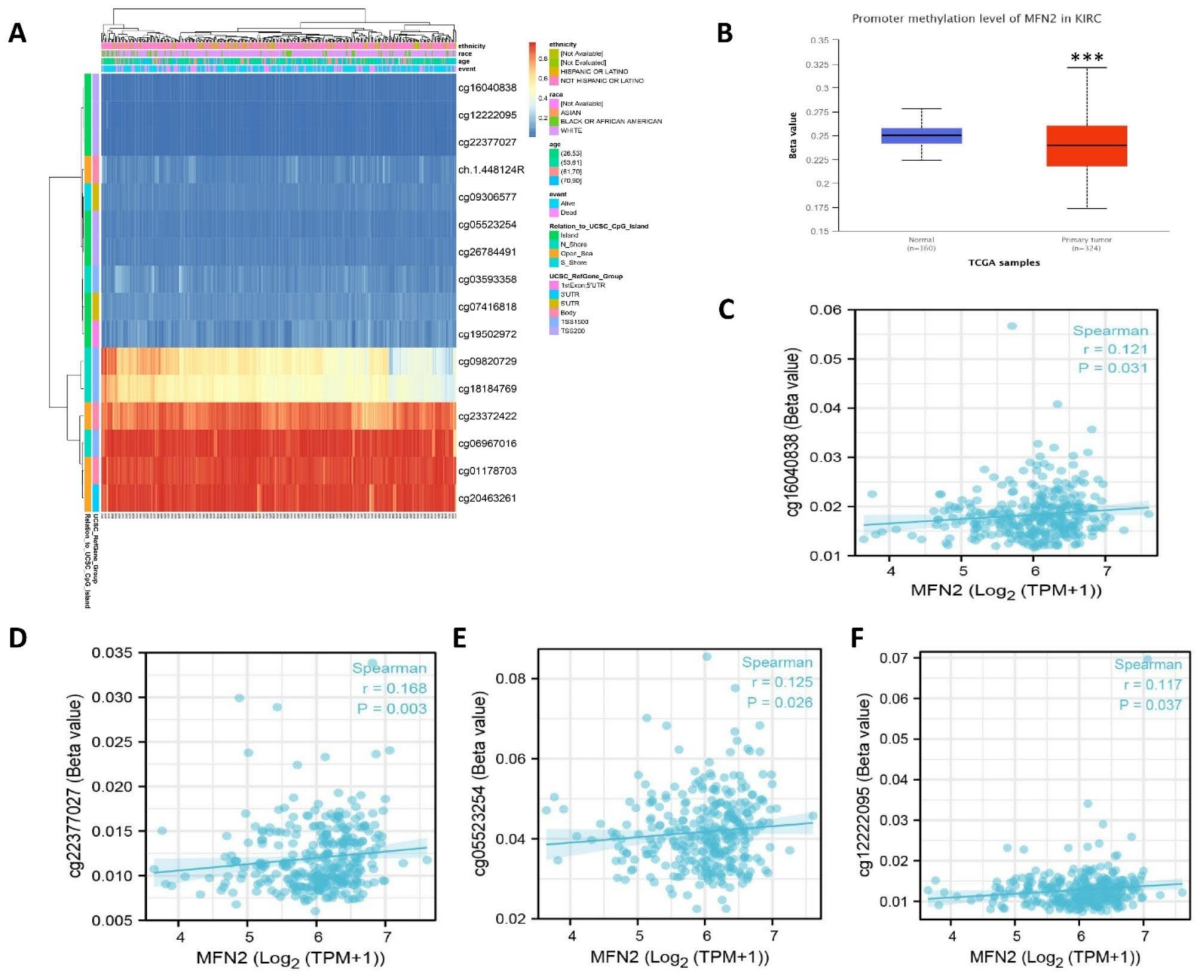
Correlation between MFN 2 expression and methylation. (**A**) Correlation between MFN2 mRNA expression level and methylation level. (**B**) The promoter methylation level of MFN2 in renal clear cell carcinoma was obtained from the UALCAN database. (**C–F**) MFN2 expression was positively correlated with methylation sites, including cg16040838, cg22377027, cg05523254, cg12222095

First, we found that most of the methylation sites in the MFN2 DNA sequence in renal clear cell carcinoma showed hypomethylation(p < 0.001)(Fig. 6A).In addition we found significantly lower levels of DNA methylation in renal clear cell carcinoma tissue than in normal kidney tissue according to UALCAN database (p < 0.001) (Fig. 6B).We also showed that MFN2 expression was positively correlated with several methylation sites, such as: cg16040838, cg22377027, cg05523254, cg12222095 (Fig. 6C-F).We also identified a number of methylation sites associated with prognosis, including cg20463261, cg01178703, cg23372422, cg06967016, cg18184769, cg05523254, cg12222095, cg22377027 and cg26784491 (Supplementary Fig. 1A-J).

### 3.7 The relationship between MFN2 expression and immune infiltration

**Fig. 7:**
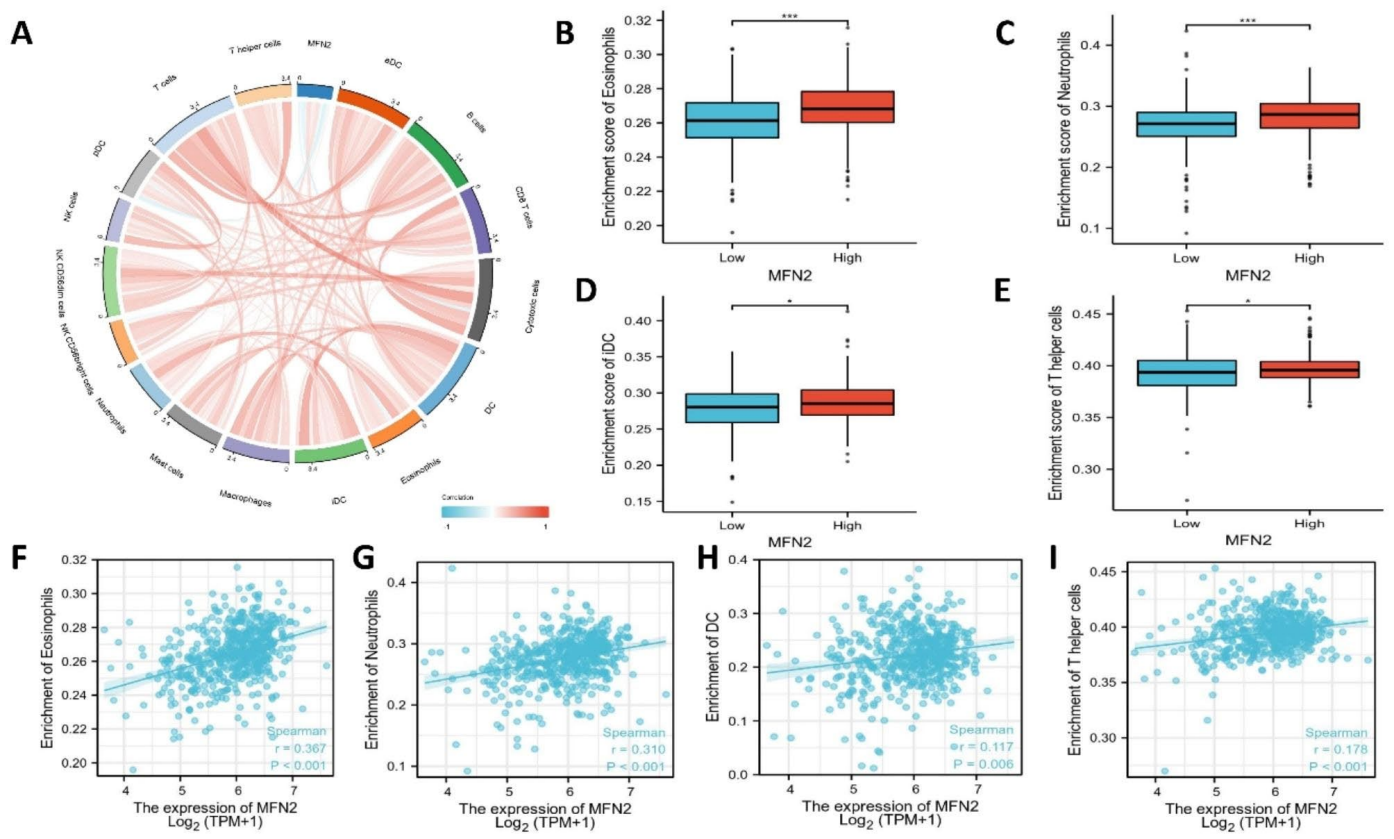
The relationship between MFN2 expression and immune infiltration in clear cell renal cell carcinoma. (**A**) Correlation between MFN2 expression and relative abundance of immune cells. Blue and red colors represent negative and positive correlations, respectively. (**B–E**) Comparison of immune infiltration levels of immune cells (including Neutrophils, Mast cells, Dendritic Cells and T helper cells) between the high- and low-MFN2 expression groups. (**F–I**) Correlations between the relative enrichment scores of immune cells (including Neutrophils, Mast cells, Dendritic Cells and T helper cells) and the expression of MFN2

MFN2 expression correlated positively with the level of immune cell infiltration in Eosinophils (r = 0.367, p < 0.001), Neutrophils (r = 0.310, p < 0.001), Mast cells (r = 0.273, p < 0.001), Dendritic Cells (r = 0.117, p = 0.006) and T helper cells (r = 0.178, p < 0.001) (Fig. 7A).The enrichment scores of Neutrophils, Mast cells, Dendritic Cells and T helper cells in the MFN2 low expression group were significantly lower than those in the MFN2 high expression group (all p < 0.01) (Fig. 7B-I).

### Prognostic value of MFN2 in the treatment of clear cell renal cell carcinoma

**Fig. 8:**
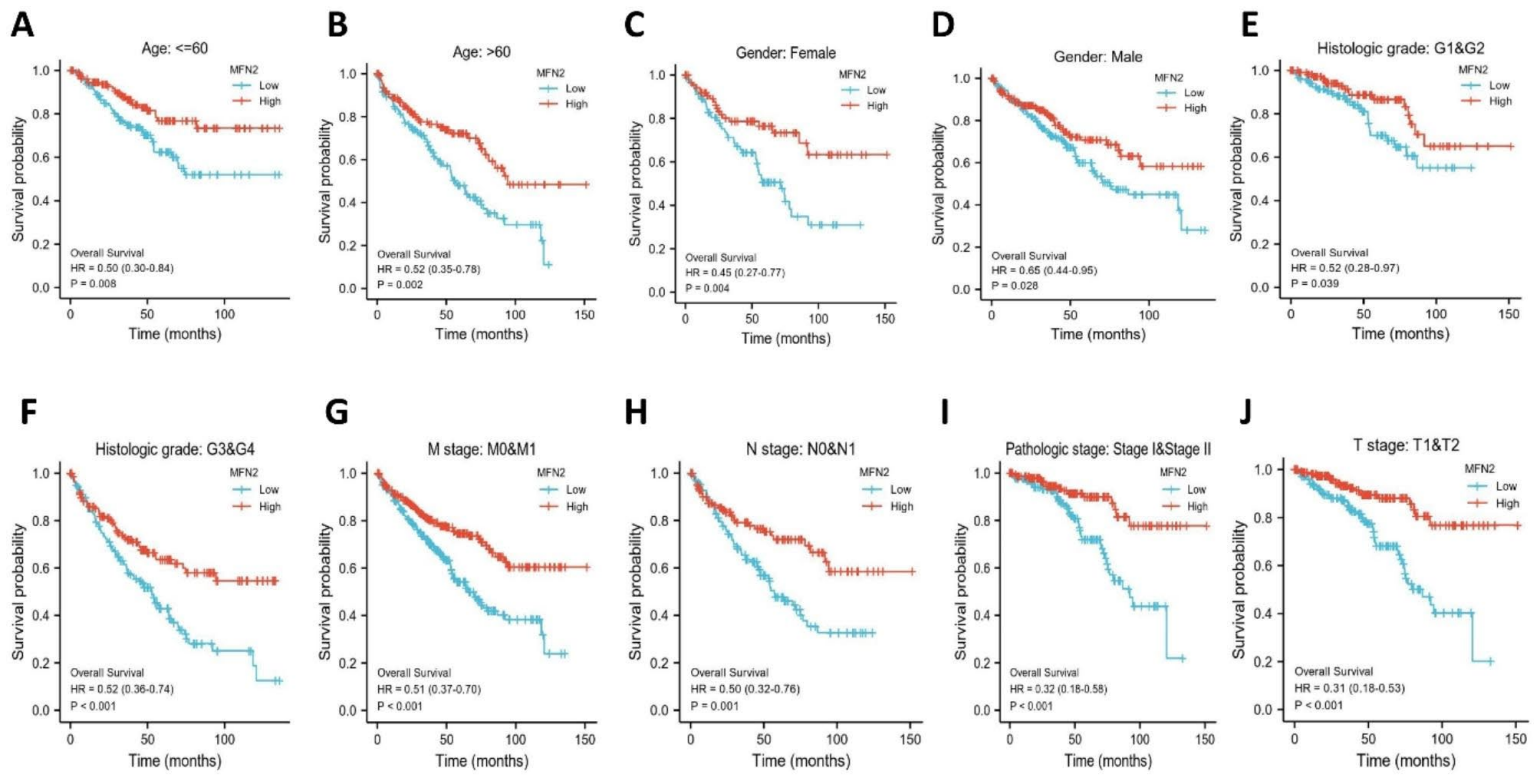
Prognostic values of MFN2 expression in patients with renal clear cell carcinoma evaluated by the Kaplan-Meier method in different subgroups. (**A–J**) OS survival curves of age < = 60 years, age > 60 years, gender, G1 and G2, G3 and G4, M0 and M1,N0 and N1, stage I and II, T1 and T2 subgroups between high- and low-MFN2 patients with renal clear cell carcinoma. OS, overall survival

For overall survival in clear cell renal cell carcinoma, low expression of MFN2 showed worse prognosis in several subgroups including age < = 60 years, age > 60 years, gender, G1 and G2, G3 and G4, M0 and M1,N0 and N1, stage I and II, T1 and T2 subgroups (all P < 0.05) (Fig. 8A-J).In addition renal clear cell carcinoma with DSS and PFI, low MFN2 expression also showed a poorer prognosis in all the above subgroups (Supplementary Fig. 2A-T).We performed univariate and multifactorial COX regression analyses on multiple variables, and multifactorial COX regression analyses demonstrated MFN2 expression (HR = 0.589,95% CI = 0.375–0.925, p = 0.021), age (HR = 1.700,95% CI = 1.110–2.605, p = 0.015), histological grade (HR = 1.660,95%CI = 1.004–2.744, p = 0.048) and M stage (HR = 2.593,95%CI = 1.530–4.397, p < 0.001) were independent prognostic indicators of OS in patients with renal clear cell carcinoma (Table 3).

**Table 3:** Univariate and multivariate analyses of MFN2 and clinicopathologic characteristics associated with OS in ccRCC patients from TCGA

Characteristics	Univariate analysis		Multivariate analysis	
	Hazard ratio (95% CI)	P value	Hazard ratio (95% CI)	P value
Age	1.765 (1.298–2.398)	**< 0.001**	1.700 (1.110–2.605)	**0.015**
Pathologic stage	3.946 (2.872–5.423)	**< 0.001**	1.252 (0.495–3.162)	0.635
Histologic grade	2.702 (1.918–3.807)	**< 0.001**	1.660 (1.004–2.744)	**0.048**
T	3.228 (2.382–4.374)	**< 0.001**	1.518 (0.666–3.462)	0.321
N	3.453 (1.832–6.508)	**< 0.001**	1.563 (0.777–3.143)	0.210
M	4.389 (3.212–5.999)	**< 0.001**	2.593 (1.530–4.397)	**< 0.001**
MFN2	0.484 (0.353–0.664)	**< 0.001**	0.589 (0.375–0.925)	**0.021**

### Construction and validation of the nomogram based on MFN2 and clinicopathological variables

**Fig. 9:**
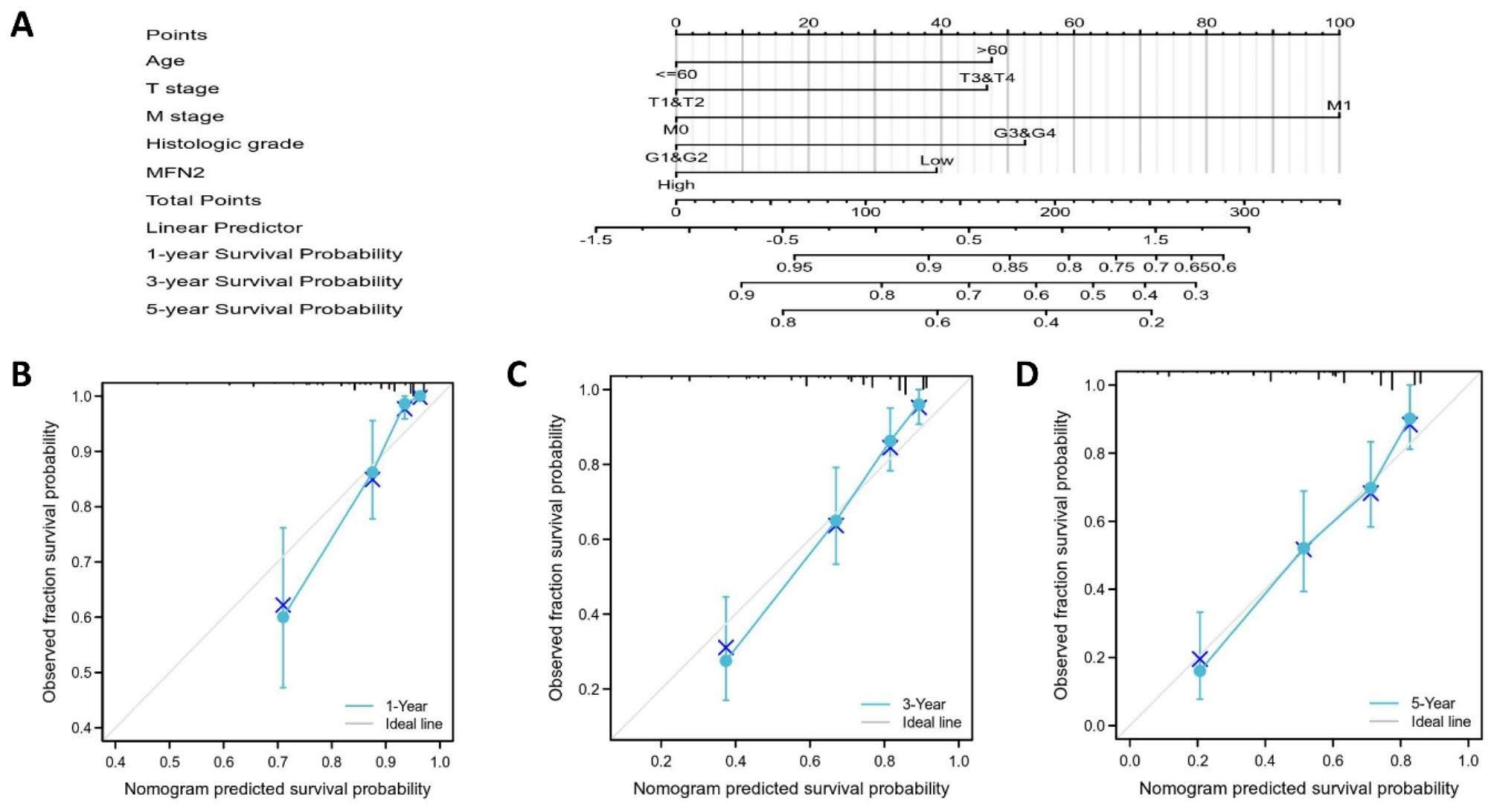
A nomogram and calibration curves for prediction of one-, three-, and five-year overall survival rates of patients with renal clear cell carcinoma. (**A**) A nomogram for prediction of one-, three-, and five -overall survival rates of patients with renal clear cell carcinoma. (**B–D**) Calibration curves of the nomogram prediction of one-, three-, and fiveyear overall survival rates of patients with renal clear cell carcinoma

Based on and OS-related independent prognostic factors, we plotted nomogram with a corrected C-index of 0.761 (Fig. 9A).In addition, we further verified the prediction effect of the nomogram by drawing the calibration curves at 1, 3, and 5 years (Fig. 9B-D).The above results indicate that this nomogram has a satisfactory ability in predicting prognosis for patients with clear cell renal cell carcinoma.

### Relationship between MFN2 and PPI in renal clear cell carcinoma

**Fig. 10:**
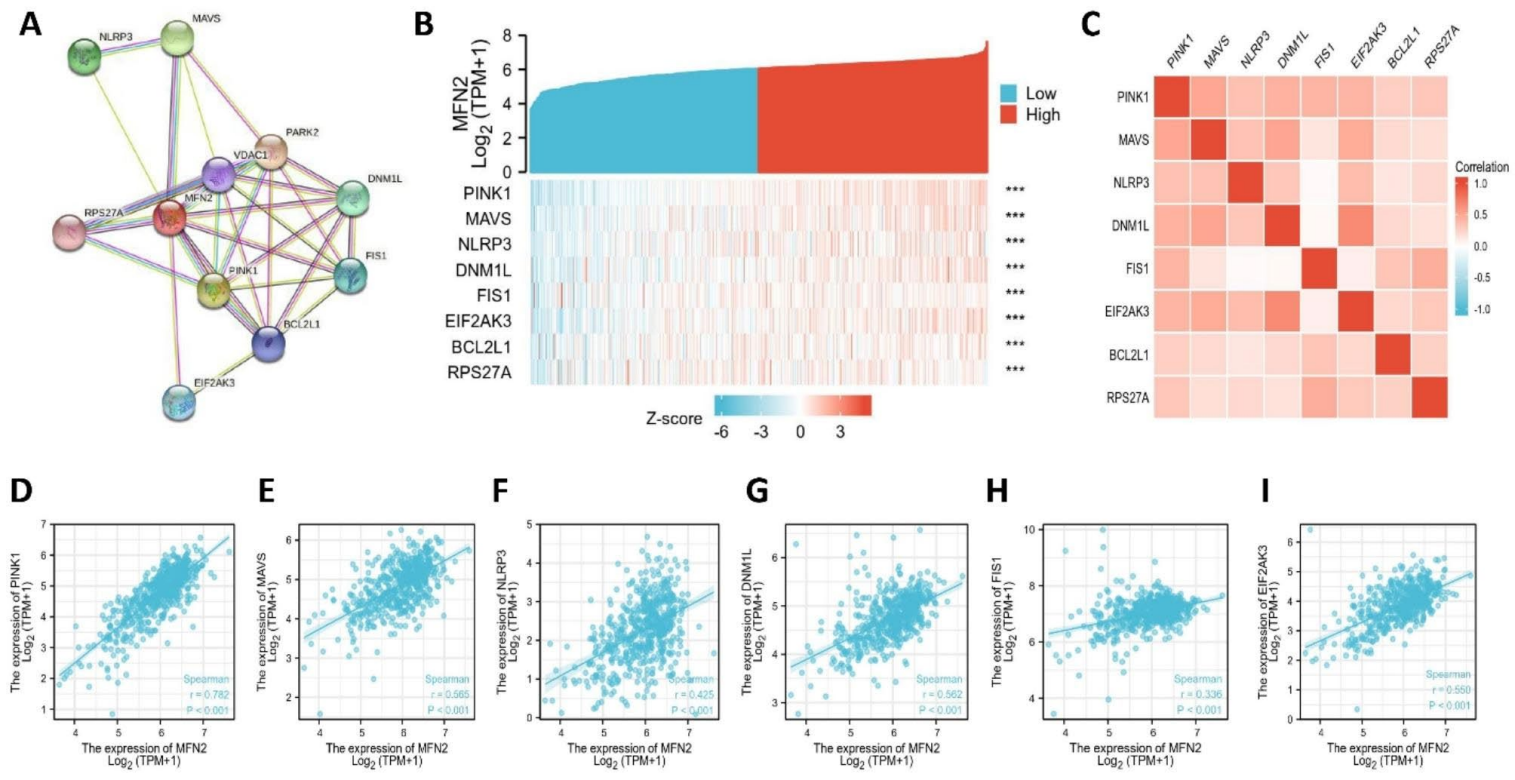
Relationship between MFN 2 and PPI in renal clear cell carcinoma. (**A**) The PPI network analysis of MFN2 in the STRING database. (**B**) Heatmap of correlation analysis of the interactive genes (including PINK1, MAVS, NLRP3, DNM1L, FIS1, EIF2AK3, BCL2L1, RPS27A) and MFN2 (all P < 0.001). (**C**) Heat map of the correlation of the interacting genes. (**D–I**) The expression of MFN2 was found to correlate with the expression of the genes PINK1 (r = 0.782, p < 0.001), MAVS (r = 0.565, p < 0.001), NLRP3 (r = 0.425, p < 0.001), DNM1L (r = 0.562, p < 0.001), FIS1 (r = 0.336, p < 0.001), and The expression of EIF2AK3 (r = 0.550, p < 0.001) was positively correlated

To further investigate genes with relevant interactions for MFN2 in renal clear cell carcinoma, we performed a PPI network analysis of MFN2 in the STRING database (Fig. 10A).We plotted the correlation analysis of the interactive genes (including PINK1, MAVS, NLRP3, DNM1L, FIS1, EIF2AK3, BCL2L1, RPS27A) and MFN2 (all P < 0.001) (Fig. 10B) and represented the correlations of the interactive genes in a heat map (Fig. 10C).The expression of MFN2 was found to correlate with the expression of the genes PINK1 (r = 0.782, p < 0.001), MAVS (r = 0.565, p < 0.001), NLRP3 (r = 0.425, p < 0.001), DNM1L (r = 0.562, p < 0.001), FIS1 (r = 0.336, p < 0.001), and The expression of EIF2AK3 (r = 0.550, p < 0.001) was positively correlated (Fig. 10D-I).

### MFN2 inhibits the proliferation and migration of renal cancer cells

**Fig. 11:**
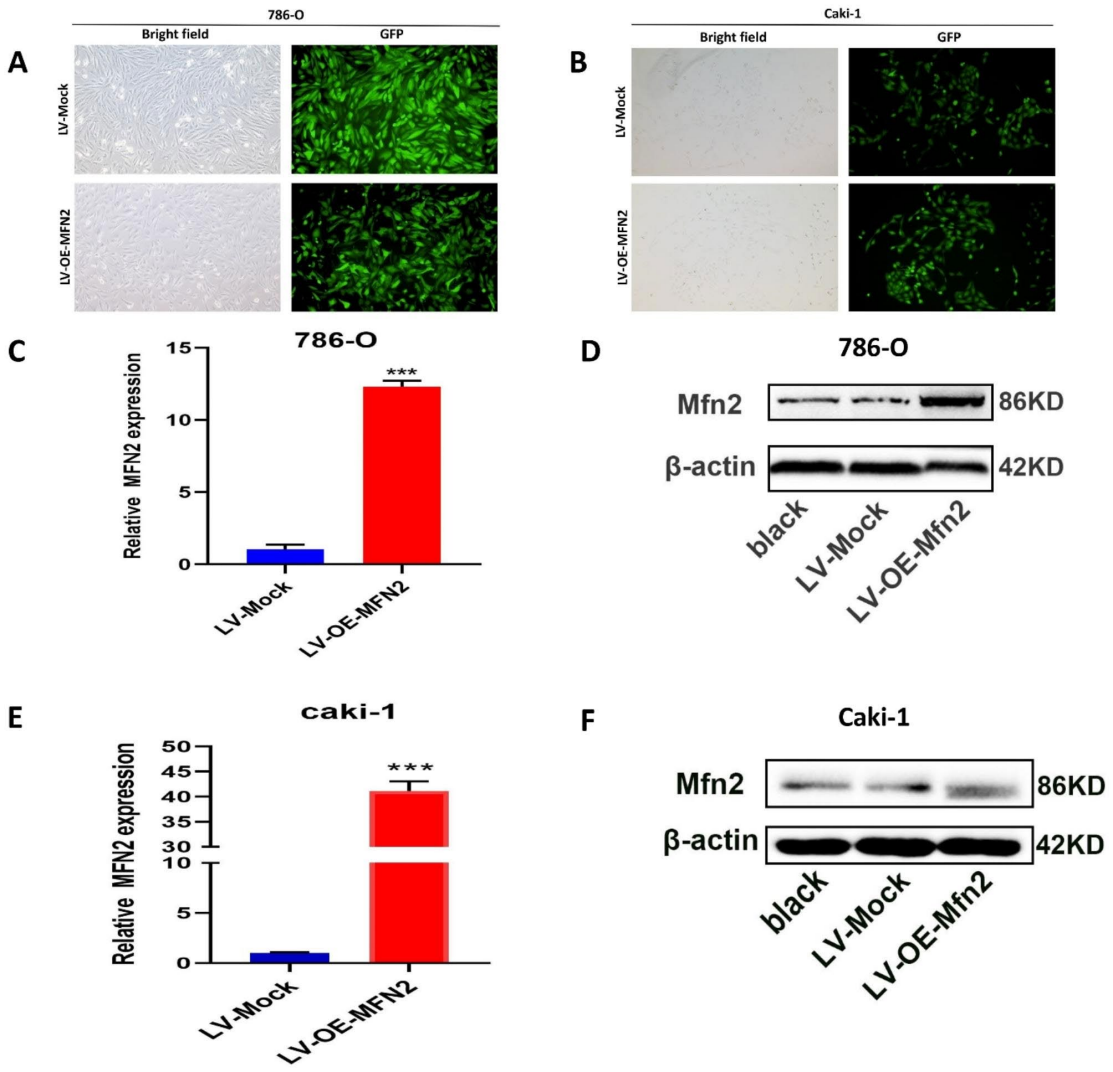
Validation of the overexpression of MFN 2 in 786O and Caki-1 cells. (**A–B**) Fluorescence photomicrographs for the experimental cell groups in 786O and Caki-1 cells after lentivirus transfection72 h. (**C–F**) The expression of MFN2 in 786O and Caki-1 cells after transfected by LV-OE-Mfn2 and LV-Mock lentivirus, respectively

**Fig. 12:**
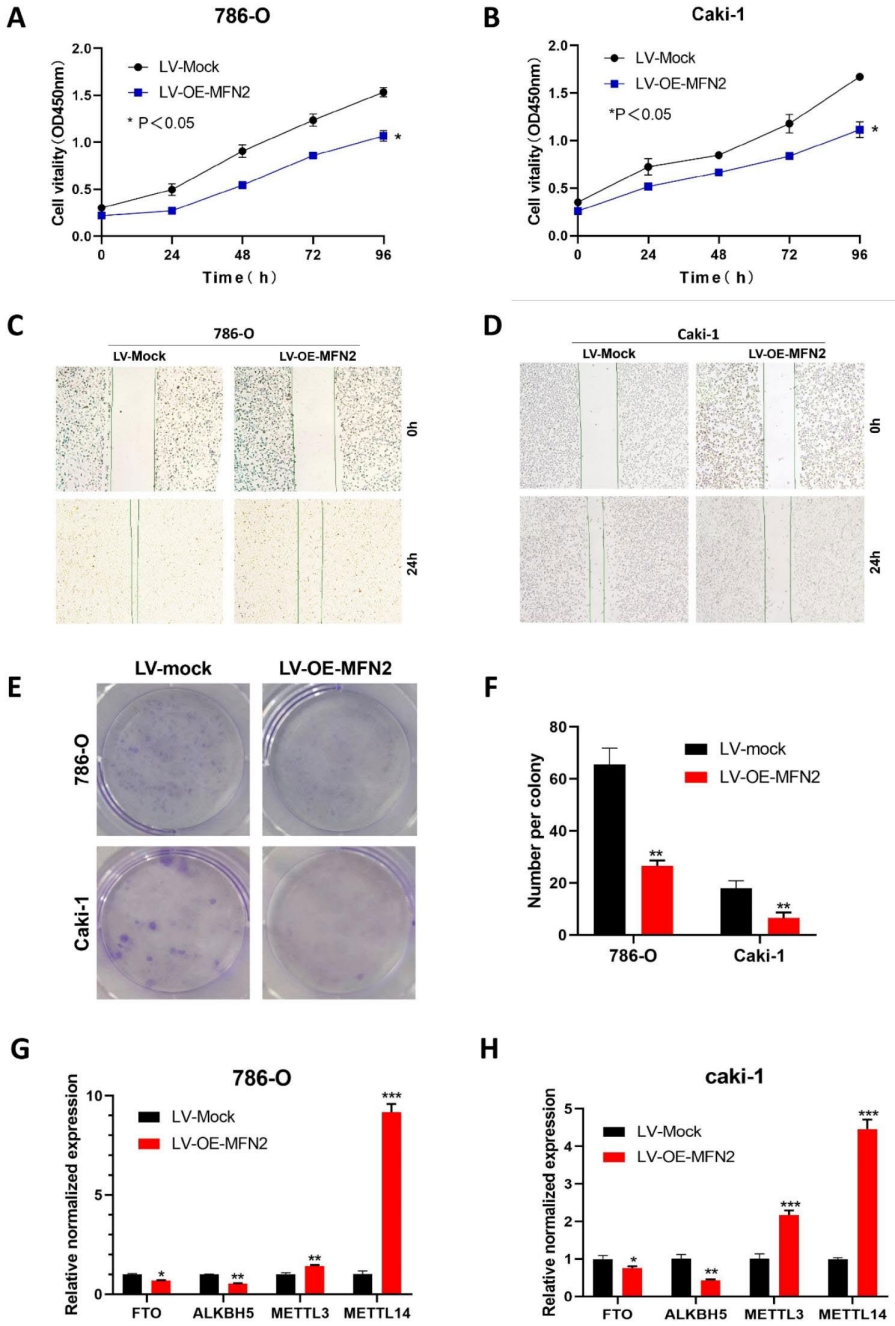
MFN 2 inhibits the proliferation and migration of renal cancer cells. (**A–B**) The CCK8 assay was performed showing that overexpression of MFN2 significantly inhibited the proliferative capacity of 786O and Caki-1 cells. (**C–D**) Wound-healing assays showed that overexpression of MFN2 inhibited the migration of 786O and Caki-1 cells. (**E–F**) Clone formation assays indicated that overexpression of MFN2 significantly inhibited the number of clone formation in 786O and Caki-1 cells. (**G–H**) The qPCR results showed that overexpression of MFN2 in 786O and Caki-1 cells decreased the expression of demethylases FTO and ALKBH5 (P < 0.05) and increased the expression of methylases METTL3 and METTL14 (P < 0.001)

To further clarify the role of MFN2 in kidney cancer cells, we subjected 786O and Caki-1 cells with low MFN2 expression to overexpression experiments.After verifying the successful transfection (Fig. 11A-B), stable transfected cell lines were established, RNA and total protein were extracted, and the overexpression efficiency of MFN2 was verified by RTPCR and WB assays both suggesting successful overexpression (Fig. 11C-F).The CCK8 assay was first performed showing that overexpression of MFN2 significantly inhibited the proliferative capacity of 786O and Caki-1 cells (Fig. 12A-B). Wound-healing assays showed that overexpression of MFN2 inhibited the migration of 786O and Caki-1 cells (Fig. 12C-D). Clone formation assays indicated that overexpression of MFN2 significantly inhibited the number of clone formation in 786O and Caki-1 cells (Fig. 12E-F).The qPCR results showed that overexpression of MFN2 in 786O and Caki-1 cells decreased the expression of demethylases FTO and ALKBH5 (P < 0.05) and increased the expression of methylases METTL3 and METTL14 (P < 0.001) (Fig. 12G-H).

## Discussion

With the advancement of precision tumour therapy, new biological markers are urgently needed for definitive diagnosis and precise treatment [29, 30].In this study, analysis of the TCGA database clarified that MFN2 was significantly low expressed in renal clear cell carcinoma relative to normal kidney tissue.It has been shown that MFN2 shows low expression in breast cancer, and MFN2 can inhibit mTORC2 expression and inhibit tumor growthby binding to mTORC2 domain HR1 [31] .A study on gastric cancer showed that the expression of MFN2 was lower than that in normal gastric mucosa tissue, and after overexpressed MFN2, it downregulated the expression of MMP-2 and MMP-9 attenuated the invasion and migration ability of cancer cells by inhibiting PI3K/Akt signaling and inhibited tumor progression [32].MFN2 regulates mitochondrial fusion / division in cells in thyroid cancer and affects cellular metabolism, which regulates EMT in tumors through induction of the AKT signaling pathway [33].In ovarian cancer, increased expression of MFN2 triggers AMPK, promotes autophagy, reduces ROS, and suppresses ovarian cancer progression through downregulation of p-mTOR and p-ERK axis [34] .MFN2 is highly expressed in cervical cancer, and the knockout of MFN2 can significantly inhibit the proliferation and EMT of cervical cancer cells, becoming a new target for the treatment of tumors [35].MFN2 expression was significantly downregulated in bladder cancer cells, and it can inhibit the Wnt/β-catenin signaling pathway to inhibit tumor progression through [36].MFN2 induces autophagy and promotes apoptotic in pancreatic cancer cells by inhibiting the PI3K/Akt/mTOR signaling pathway in pancreatic cancer [37].However, how MFN2 is expressed and how it functions in renal clear cell carcinoma is unclear and requires further study.In our study, MFN2 showed low expression and correlated with worse pathological stage, T-stage, histological grade, and M-stage.In addition, we found that low expression of MFN2 was associated with poorer OS, DSS and PFI, and univariate and multifactorial COX regression analyses showed that MFN2 expression was an important independent prognostic factor for renal clear cell carcinoma. Taken together, MFN2 could be a new molecular candidate to treat renal clear cell carcinoma.

Mitofusin 2 is mainly found on the outside mitochondrial membrane and its main function is to participate in mitochondrial fusion. Tumour cells are highly heterogeneous and aggressive, with a high proliferative capacity, and during cell proliferation, mitochondrial division increases and fusion decreases, which is the reason for the low expression of MFN2 in renal clear cell carcinoma in our analysis.During our pathway enrichment of MFN2, we discovered that low expression of MFN2 was negatively and significantly associated with the G2-M DNA damage checkpoint and oxidative stress-induced senescence, which may be the mechanism by which it exerts its oncogenic effects.Previous studies have reported that MFN2-mediated mitochondrial fusion inhibits ovarian cancer invasion by attenuating ROS and promoting autophagy.Whether MFN2 has the same biological function in renal clear cell carcinoma requires us to further verify.Previous studies have shown that low MFN2 expression in breast cancer is associated with its hypermethylation at the promoter, and when demethylated breast cancer cells were treated, MFN2 expression was detected to be increased in a dose-dependent way, and its anti-tumour mechanism may be related to DNA hypermethylation at the promoter [38].Our results showed that MFN2 showed hypomethylation status in renal clear cell carcinoma, but also some methylation sites showed hypermethylated expression. After overexpression of MFN2, the expression of methylase was increased and the expression of demethylase was decreased.This is different from the previous expression in breast cancer, and we analyze that it may be related to various reasons such as tumor type or ethnic population, and the exact mechanism of action needs to be clarified by our further study.

Immune cells are present in the tumour cell microenvironment and often encase tumour cells, and the role of tumour cells in tumour development through immune infiltration has been reported [39].Screening of invasive immune cells has important implications for immune checkpoint therapy of tumours.We found that MFN2 expression was positively correlated with a variety of immune cells, such as Mast cells, Dendritic Cells and T helper cells.It has been shown that killer cells combined with dendritic cells can be used to treat renal cell carcinoma with good results [40].This further suggests that low expression of MFN2 can influence the prognosis of renal cell carcinoma by regulating infiltrating immune cells.

We have verified in vitro cell experiments to correlate the lower level of MFN2 expression with the ability of renal clear cell carcinoma cells to proliferate and migrate.However, our study still has some limitations. Firstly, we only verified the expression of MFN2 and its correlation with prognosis through the TCGA database, which may have some bias; secondly, we need to further elucidate how MFN2 plays a role in renal clear cell carcinoma through in vivo experiments and in vitro mechanistic experiments.

## Conclusions

In summary, we verified through the present research that low MFN2 expression is an important independent prognostic element in renal clear cell carcinoma and is significantly correlated with the proliferation and migration ability of the tumour.MFN2 can be a new prognostic marker for renal clear cell carcinoma patients.After that, we need to further investigate the mechanism of action of MFN2 in renal clear cell carcinoma.

### Electronic supplementary material

Below is the link to the electronic supplementary material.

Supplementary Material 1

Supplementary Material 2

Supplementary Material 3

Supplementary Material 4

Supplementary Material 5

## Data Availability

Raw data may be requested from the corresponding author with the permission of the institution.

## Abbreviations

MFN2: Mitofusin 2
TCGA: The Cancer Genome Atlas
RCC: Renal Cell Carcinoma
ccRCC: Clear Cell Renal Cell Carcinoma
GTEx: Genotype-Tissue Expression
GEO: Gene Expression Omnibus
DEGs: Differentially Expressed Genes
GO: Gene Ontology
KEGG: Kyoto Encyclopedia of Genes and Genomes
GSEA: Gene Set Enrichment Analysis
OS: Overall Survival
DSS: Disease Specific Survival
PFI: Progression-Free Interval
PPI: Protein-Protein Interaction Networks
STRING: Search Tool for the Retrieval of Interacting Genes
ROS: Receiver Operating Characteristic Curve
