# Supplementary material for: The mitochondrial fusion-associated protein MFN2 can be used as a novel prognostic molecule for clear cell renal cell carcinoma
Source: BMC Cancer. 2023 Oct 16;23:986. doi: 10.1186/s12885-023-11419-8 (PMC10577979; doi:10.1186/s12885-023-11419-8)
Supplement: Supplementary file 1 — Supplementary Material 1 [file 12885_2023_11419_MOESM1_ESM.docx]

Supplementary Table S1 The relationship of MFN2 with the first 10 DEGs

| Target molecules | Other molecules | correlation coefficent  (Pearson) | P value  (Pearson) | correlation coefficent  (Spearman) | P value  (Spearman) |
| --- | --- | --- | --- | --- | --- |
| MFN2 | SLC12A3 | 0.206 | <0.001 | 0.194 | <0.001 |
| MFN2 | SLC13A2 | 0.252 | <0.001 | 0.363 | <0.001 |
| MFN2 | SLC12A1 | 0.238 | <0.001 | 0.258 | <0.001 |
| MFN2 | CASP14 | 0.071 | 0.100 | -0.093 | 0.030 |
| MFN2 | SOSTDC1 | 0.383 | <0.001 | 0.425 | <0.001 |
| MFN2 | CTXN3 | 0.213 | <0.001 | 0.234 | <0.001 |
| MFN2 | PASD1 | 0.117 | 0.007 | -0.112 | 0.009 |
| MFN2 | AC117457.1 | 0.170 | <0.001 | 0.195 | <0.001 |
| MFN2 | ELMOD1 | 0.208 | <0.001 | 0.115 | 0.007 |
| MFN2 | AQP2 | 0.153 | <0.001 | 0.098 | 0.022 |
