# Supplementary material for: The mitochondrial fusion-associated protein MFN2 can be used as a novel prognostic molecule for clear cell renal cell carcinoma
Source: BMC Cancer. 2023 Oct 16;23:986. doi: 10.1186/s12885-023-11419-8 (PMC10577979; doi:10.1186/s12885-023-11419-8)
Supplement: Supplementary file 2 — Supplementary Material 2 [file 12885_2023_11419_MOESM2_ESM.docx]

Supplementary Table S2 GO analysis of DEGs

| Ontology | ID | Description | GeneRatio | BgRatio | pvalue | p.adjust | qvalue |
| --- | --- | --- | --- | --- | --- | --- | --- |
| BP | GO:0070268 | cornification | 14/179 | 112/18670 | 3.53e-12 | 6.22e-09 | 5.94e-09 |
| BP | GO:0006953 | acute-phase response | 9/179 | 47/18670 | 5.57e-10 | 4.92e-07 | 4.70e-07 |
| CC | GO:0000786 | nucleosome | 16/185 | 107/19717 | 3.86e-15 | 6.87e-13 | 5.12e-13 |
| CC | GO:0044815 | DNA packaging complex | 16/185 | 115/19717 | 1.25e-14 | 1.11e-12 | 8.29e-13 |
| MF | GO:0031492 | nucleosomal DNA binding | 6/141 | 55/17697 | 4.83e-06 | 5.74e-04 | 5.10e-04 |
| MF | GO:0004252 | serine-type endopeptidase activity | 9/141 | 160/17697 | 5.45e-06 | 5.74e-04 | 5.10e-04 |
