# Supplementary material for: The mitochondrial fusion-associated protein MFN2 can be used as a novel prognostic molecule for clear cell renal cell carcinoma
Source: BMC Cancer. 2023 Oct 16;23:986. doi: 10.1186/s12885-023-11419-8 (PMC10577979; doi:10.1186/s12885-023-11419-8)
Supplement: Supplementary file 3 — Supplementary Material 3 [file 12885_2023_11419_MOESM3_ESM.docx]

Supplementary Table S3 KEGG analysis of DEGs

| Ontology | ID | Description | GeneRatio | BgRatio | pvalue | p.adjust | qvalue |
| --- | --- | --- | --- | --- | --- | --- | --- |
| KEGG | hsa05322 | Systemic lupus erythematosus | 13/57 | 136/8076 | 6.39e-12 | 6.26e-10 | 5.92e-10 |
| KEGG | hsa05034 | Alcoholism | 14/57 | 187/8076 | 2.52e-11 | 1.24e-09 | 1.17e-09 |
| KEGG | hsa05203 | Viral carcinogenesis | 7/57 | 204/8076 | 5.36e-04 | 0.018 | 0.017 |
