# Supplementary material for: The mitochondrial fusion-associated protein MFN2 can be used as a novel prognostic molecule for clear cell renal cell carcinoma
Source: BMC Cancer. 2023 Oct 16;23:986. doi: 10.1186/s12885-023-11419-8 (PMC10577979; doi:10.1186/s12885-023-11419-8)
Supplement: Supplementary file 4 — Supplementary Material 4 [file 12885_2023_11419_MOESM4_ESM.pdf]

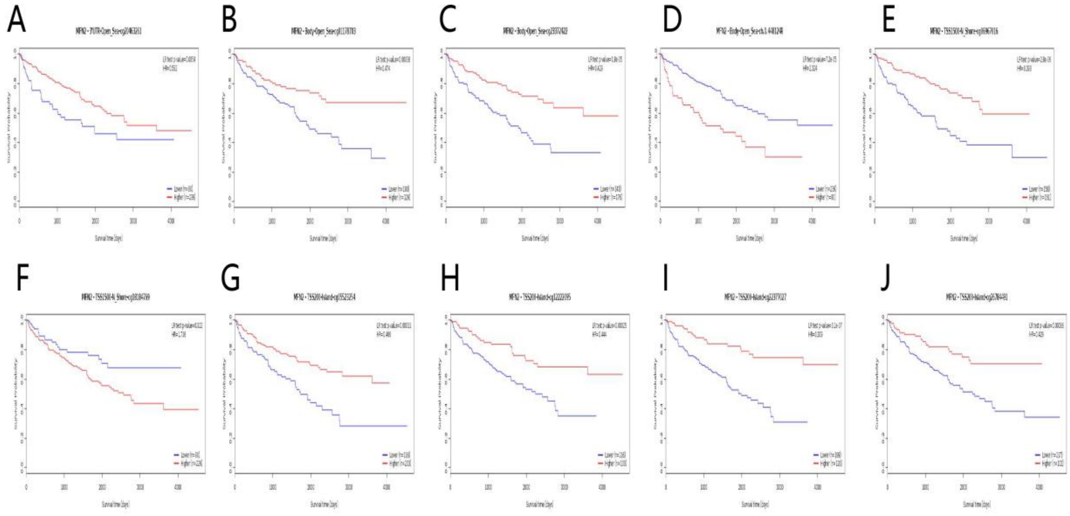

Supplementary Figure 1 The impact of DNA methylation levels of MFN2 on the prognosis of patients with renal clear cell carcinoma.(A-J)Kaplan-Meier survival curves for several methylation sites of MFN2,including cg20463261, cg01178703, cg23372422, cg06967016, cg18184769, cg05523254, cg12222095, cg22377027 and cg26784491.

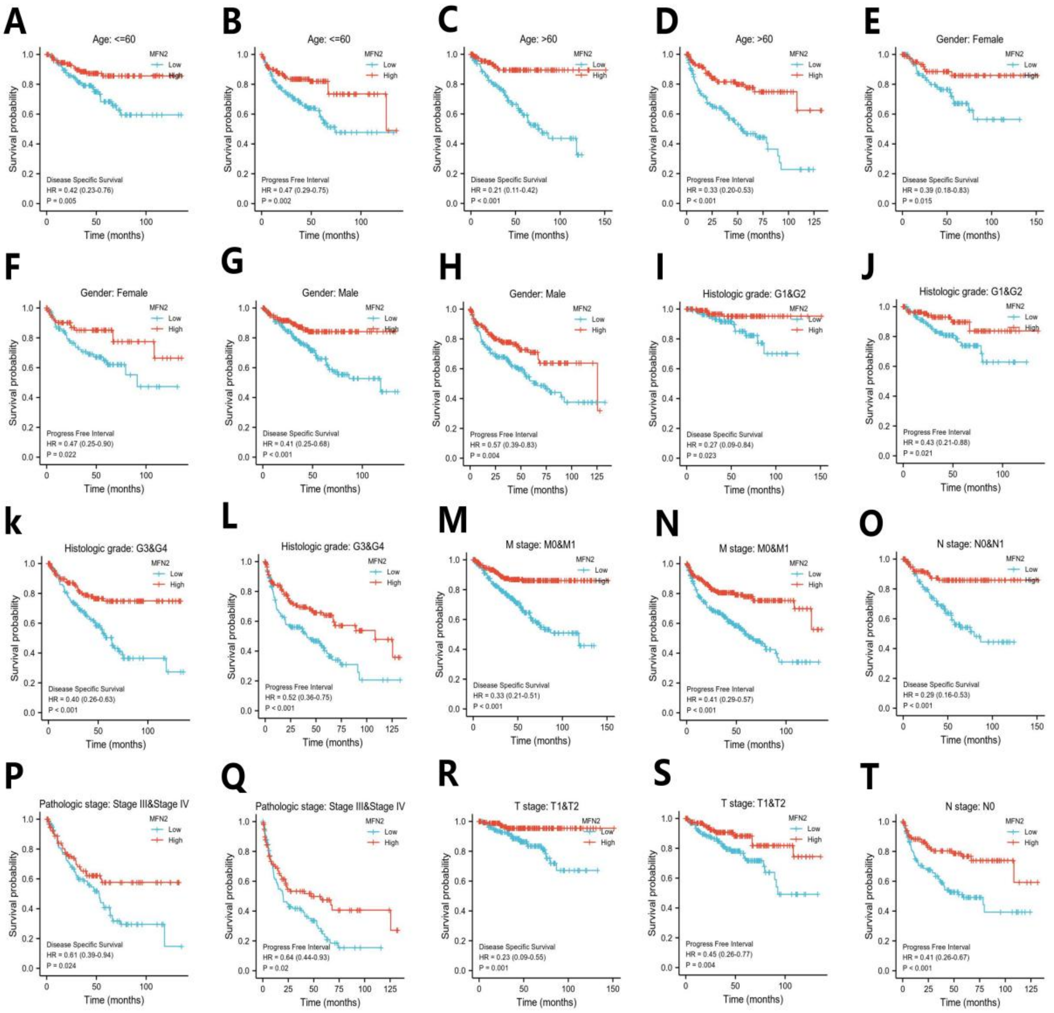

Supplementary Figure 2 Prognostic values of MFN2 expression in patients with renal clear cell carcinoma evaluated by the Kaplan-Meier method in different subgroups.(A-T)DSS and PFI survival curves of age <= 60 years, age > 60 years, gender, G1 and G2, G3 and G4, M0 and M1,N0 and N1, stage I and II, T1 and T2 subgroups between high- and lowMFN2 patients with renal clear cell carcinoma.DSS, disease specific survival;PFI,Progress free interval.

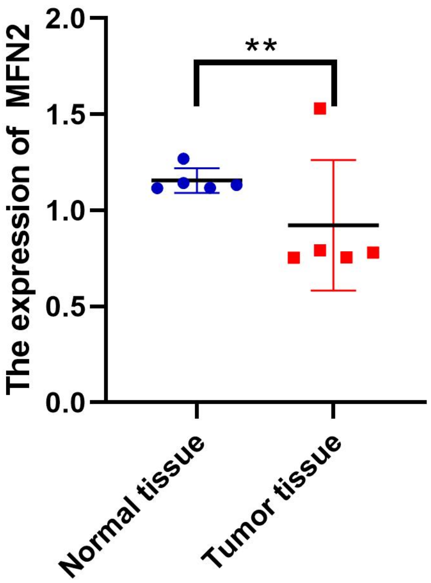

Supplementary Figure 3 Proteomic data of carcinomas and adjacent cancers from 5 renal clear cell carcinoma patients in our center, MFN 2 was low expressed in tumor tissue (P <0.01).
