# Supplementary material for: The mitochondrial fusion-associated protein MFN2 can be used as a novel prognostic molecule for clear cell renal cell carcinoma
Source: BMC Cancer. 2023 Oct 16;23:986. doi: 10.1186/s12885-023-11419-8 (PMC10577979; doi:10.1186/s12885-023-11419-8)
Supplement: Supplementary file 5 — Supplementary Material 5 [file 12885_2023_11419_MOESM5_ESM.pdf]

Figure2A

MFN2 Protein order： normal1、cancer1、normal2、cancer2、normal3、cancer3、normal4、cancer4

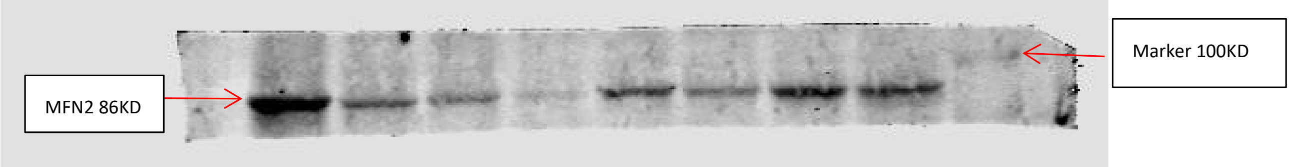

β -actin

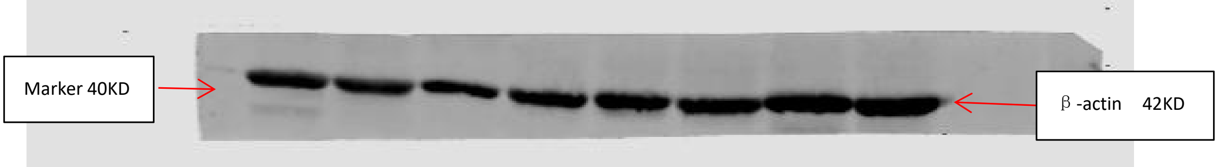

MFN2 Protein order： normal5、cancer5、normal6、cancer6、normal7、cancer7、normal8、cancer8

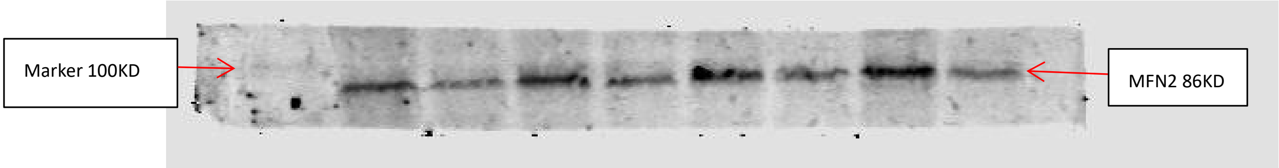

and β-actin

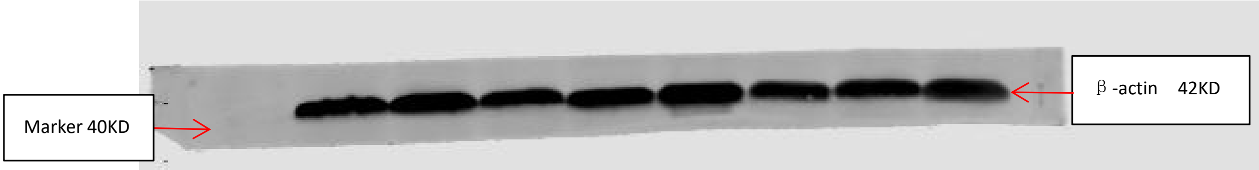

Figure2C Protein order： HK2、786O、caki-1、A498

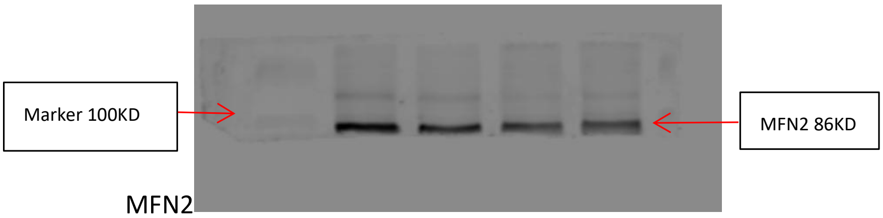

MFN2

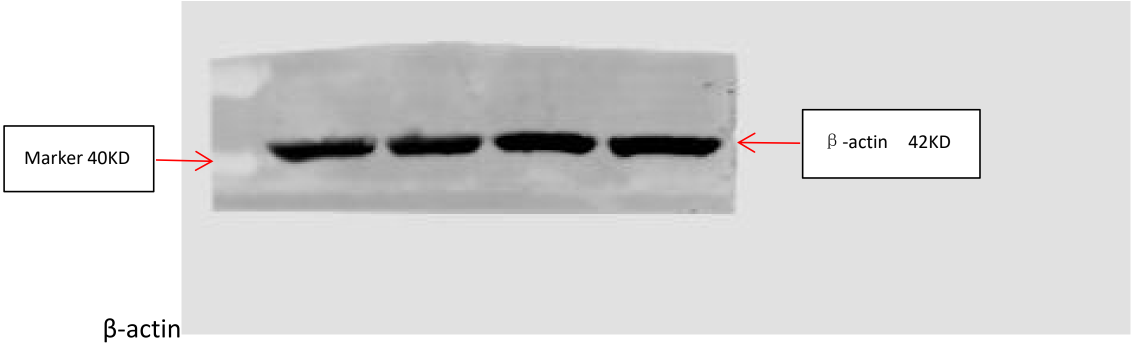

β-actin

Figure11D

MFN2 Protein order： 786O-black 、 786O-LV-Mock 、 7860-LV-OE-MFN2

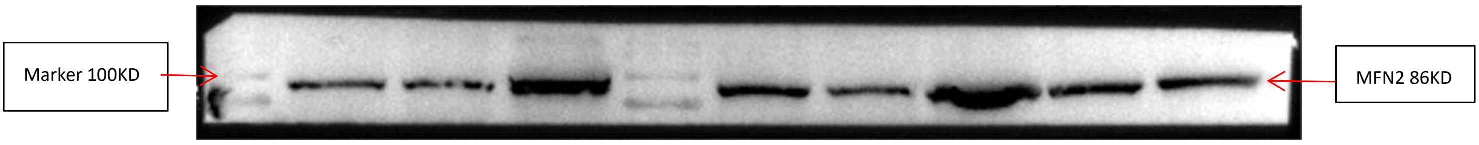

β-actin

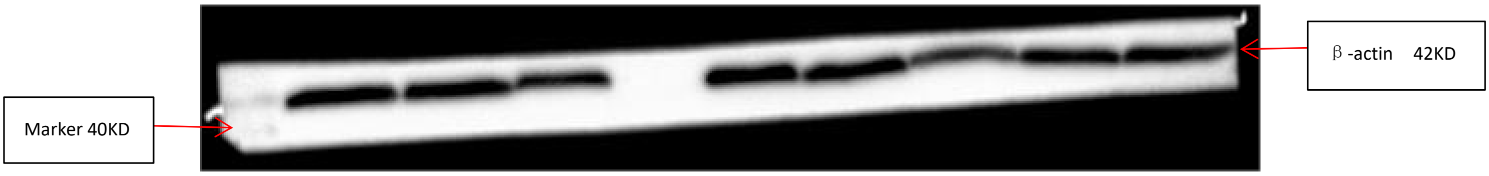

Figure11F Protein order：caki-1-black、caki-1-LV-Mock、caki-1-LV-OE-MFN2

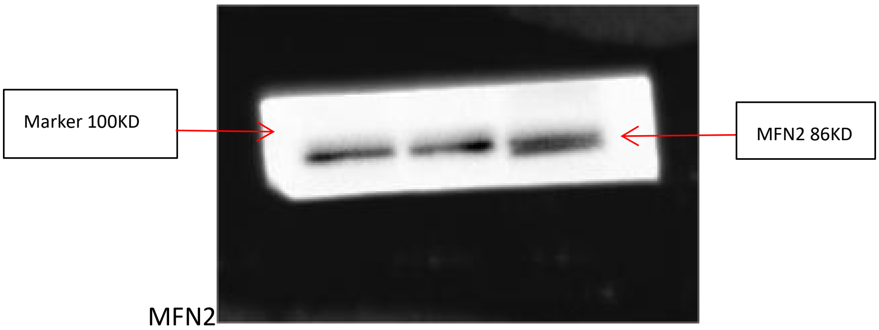

MFN2

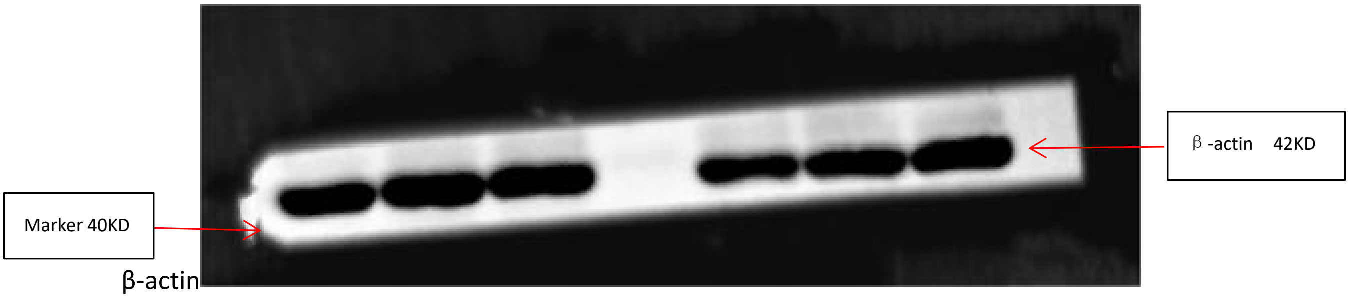

β-actin
